# Growth inhibition of *S. cerevisiae*, *B. subtilis*, and *E. coli* by lignocellulosic and fermentation products

**DOI:** 10.1007/s00253-016-7642-1

**Published:** 2016-06-04

**Authors:** Joana P. C. Pereira, Peter J. T. Verheijen, Adrie J. J. Straathof

**Affiliations:** Department of Biotechnology, Delft University of Technology, van der Maasweg 9, 2629 HZ Delft, the Netherlands

**Keywords:** Bio-based products, Growth inhibition, Lag-time model, Product-inhibition models

## Abstract

This paper describes the effect of several inhibiting components on three potential hosts for the bio-based production of methyl propionate, namely, wild-type *Escherichia coli* and *Bacillus subtilis*, and evolved *Saccharomyces cerevisiae* IMS0351. The inhibition by the lignocellulose-derived products 5-hydroxymethyl-2-furaldehyde, vanillin, and syringaldehyde and the fermentation products 2-butanol, 2-butanone, methyl propionate, and ethyl acetate has been assessed for these strains in defined medium. Multiple screenings were performed using small-scale cultures in both shake flasks and microtiter plates. Technical drawbacks revealed the limited applicability of the latter in this study. The microbial growth was characterized by means of a lag-time model, and the inhibitory thresholds were determined using product-inhibition models. The lignocellulose-derived products were found to be highly inhibitory, and none of the strains could grow in the presence of 2.0 g L^−1^ of product. From the fermentation products tested, methyl propionate had the most severe impact resulting in complete inhibition of all the strains when exposed to concentrations in the range of 12–18 g L^−1^. In general, *S. cerevisiae* and *B. subtilis* were comparatively more tolerant than *E. coli* to all the fermentation products, despite *E. coli*’s lower sensitivity towards vanillin. The results suggest that, overall, the strains investigated have good potential to be engineered and further established as hosts for the bio-based production of methyl esters.

## Introduction

Methyl methacrylate is a valuable building block for acrylic paints and organic glass (Kent 2013). The global demand for methyl methacrylate has grown annually, and it is expected to increase at an average rate of 4.0 % up to 2016 (Davis 2012). Currently, methyl methacrylate is produced from fossil feedstocks, such as methyl propionate (Li et al. 2013; Shreiber et al. 1996). Therefore, its market growth is vulnerable to rising and volatile fossil feedstock prices. The development of a bio-based production process would mitigate these effects and exploit the potential of these methyl esters. Recent findings show that methyl propionate can be formed by enzymatic oxidation of 2-butanone (van Beek et al. 2014). The fermentative production of 2-butanone has also been proposed, both in *Escherichia coli* (Yoneda et al. 2014) and *Saccharomyces cerevisiae* (Ghiaci et al. 2014). Despite the low conversion efficiencies reached so far, the coupling of these processes would enable the use of renewable feedstocks such as lignocellulose, instead of fossil feedstocks, for the long-term production of methyl methacrylate. However, in addition to demanding pathway engineering, product toxicity is a major drawback in the microbial production of commodity chemicals.

Lignocellulose is the most abundant biomass on earth, and it is the substrate of choice to produce bulk products by fermentation (Eriksson and Bermek 2009; Straathof 2014). Given its complex structure consisting of cellulose, hemicellulose, and lignin, lignocellulose requires pretreatment to facilitate depolymerization to simple sugars. Several pretreatment methods have been inspected comprising both chemical and enzymatic hydrolysis, but the unavoidable release of inhibitory degradation products is often emphasized and strongly correlated to the type of feedstock and pretreatment used (Du et al. 2010; Ibraheem and Ndimba 2013; van der Pol et al. 2014). Typical potential inhibitors include weak acids, phenolic compounds like vanillin and syringaldehyde, and furanic compounds such as 2-furaldehyde (furfural) and 5-hydroxymethyl-2-furaldehyde (HMF) (Jönsson et al. 2013; Luo et al. 2002; van der Pol et al. 2014). The effect of these compounds on the growth and productivity of different microorganisms has been reviewed by many authors, but the levels of inhibition reported vary strikingly with inhibitor concentrations and microbial strain (Larsson et al. 2000; Pienkos and Zhang 2009; van der Pol et al. 2014; Wierckx et al. 2011).

Besides lignocellulosic degradation products, fermentation products are also toxic to the fermenting microorganisms (Aiba et al. 1968; Kanno et al. 2013; Urit et al. 2013). In addition to methyl propionate, intermediates such as 2-butanone, 2-butanol, and ethyl acetate are also expected to be produced. 2-Butanone has been reported to decrease the cell density of *E. coli* and *S. cerevisiae* strains by 85 and 53 %, respectively, for concentrations around 2.5 % (*v*/*v*) (Burk et al. 2010). The inhibiting effect of different butanol isomers on the growth of *S. cerevisiae* has also been investigated (Ghiaci et al. 2013; Gonzalez-Ramos et al. 2013), and the studies report that the growth rate of *S. cerevisiae* is barely affected when growing in 2-butanol concentrations up to 1.2 % (*v*/*v*) (Ghiaci et al. 2013). Other inhibition studies have shown that the microbial growth of *Kluyveromyces marxianus* and *Hydrangea anomala* is totally inhibited by nearly 2.0 % (*v*/*v*) ethyl acetate (Tabachnick and Joslyn 1953; Urit et al. 2013). Surprisingly, the effect of methyl propionate on fermenting microorganisms has not yet been described.

The inhibition of microbial hosts by both lignocellulosic and fermentation products often leads to low yields and productivity, increasing product recovery and energy costs significantly (Oudshoorn et al. 2010). As a result, the bio-based production cannot compete economically with the chemical synthesis. Therefore, finding a user-friendly tolerant host will enhance the productivity and promote the bio-based methyl ester production.

While *E. coli* has been widely used as platform microorganism for metabolic engineering regarding 2-butanone and butanol production (Atsumi et al. 2008; Atsumi and Liao 2008; Kanno et al. 2013; Reyes et al. 2012; Yoneda et al. 2014), *S. cerevisiae* IMS0351 has already been identified as highly tolerant to alcohols (Gonzalez-Ramos et al. 2013) and *Bacillus subtilis* has been recognized as a potential platform for biocommodity production from nonfood biomass (Anderson et al. 2013; Kataoka et al. 2011; Zhang and Zhang 2010). In this paper, the inhibition of these three potential hosts by lignocellulose degradation products, namely, HMF, vanillin, and syringaldehyde, and fermentation products, namely, 2-butanol, 2-butanone, methyl propionate, and ethyl acetate, has been assessed. Multiple inhibition assays were conducted on small-scale cultures, using both shake flasks (SFs) and microtiter plates (MTPs). The maximum growth rates at high dilution and microbial lag-times were determined for each assay using the lag-time model proposed by Baranyi and Roberts (1994). The inhibitory thresholds were further assessed using known product-inhibition models (Aiba et al. 1968; Dagley and Hinshelwood 1938; Quintas et al. 2005). Based on the results, this study ultimately evaluates the potential of each microbial host for recombinant solvent production, which can enable the bio-based production of methyl propionate.

## Materials and methods

### Microbial strains and culture media

The laboratory strains *E. coli* K12 DH5α, *B. subtilis* NCCB 70064, and *S. cerevisiae* IMS0351 (Gonzalez-Ramos et al. 2013) were kindly provided by the Industrial Microbiology group, Delft University of Technology. Stock cultures were stored at −80 °C in a mixture containing fermentation media and 20 % glycerol.

The strains were grown in appropriate chemically defined mineral media: *E. coli* and *B. subtilis* were grown in medium as in Cuellar et al. (2009), and *S. cerevisiae* was grown in medium as in Verduyn et al. (1992). Fresh solutions were prepared aseptically immediately before each experiment, using 15 g L^−1^ glucose as carbon source. All the reagents used were of analytical grade.

Prior to each inhibition assay, 100 mL fermentation medium was directly inoculated with cells taken from the frozen stocks and incubated aerobically overnight at 200 rpm and appropriate temperature (37 °C for *E. coli* and *B. subtilis*; 30 °C for *S. cerevisiae*). Solutions of inhibiting agents *i* were prepared according to the concentrations *C*_*i*_ (g L^−1^) depicted in the “Results” section. The reference stands for fresh fermentation medium without any inhibitor. The initial pH of each solution was adjusted using KOH (4 mol L^−1^) and H_2_SO_4_ (2 mol L^−1^), aiming at pH 6.5 for *E. coli* and *B. subtilis* and pH 4.5 for *S. cerevisiae*. The pH was not controlled during the experiments.

### Inhibition assays in shake flasks

For manual growth measurements, 80-mL glass flasks were aseptically filled with 19 mL fresh fermentation medium containing inhibitor concentrations in the defined ranges. Each flask was inoculated with 1-mL aliquots from the overnight grown cultures to an initial OD_600_ of approximately 0.15. After inoculation, the flasks were sealed with pierceable rubber stoppers to prevent evaporation during sampling and incubated at 150 rpm in an orbital shaker with 5 cm shaking diameter and suitable temperature (37 °C ± 1 °C for *E. coli* and *B. subtilis*; 30 °C ± 1 °C for *S. cerevisiae*). The mixing performance and oxygen transfer rate (OTR) were assessed using the correlations proposed by Maier and Büchs (2001) and Klockner and Büchs (2012), and a value of 7 mmol O_2_ L^−1^ h^−1^ was found for the OTR under these conditions. The growth curves were determined by measuring the OD_600_ of each flask every 2 h during 14 h in a Biochrom Libra S11 Visible Spectrophotometer, and a final measurement was performed after 24 h. All the measurements were performed within the linear OD range of the instrument, using fresh fermentation medium for sample dilution when required. To determine whether evaporation or microbial consumption occurred throughout the experiments, the initial and final concentrations of the volatile inhibitors were determined via GC (Focus GC, Interscience, Thermo Electron), using an aqueous solution of 325 mg L^−1^ 1-pentanol as internal standard. Two independent experiments were run in duplicate.

### Inhibition assays in microtiter plates

For growth measurements in microtiter plates, 392-μL Greiner 96-well MTPs with flat bottom and low evaporation lid were used. The wells were aseptically filled with 190 μL fresh fermentation medium containing inhibitor concentrations in the defined ranges. Each well was inoculated with 10 μL from the cultures grown overnight to an initial OD_600_ of approximately 0.15, and at least 16 replicates were used per condition. Given the large amount of conditions to be tested, three similar microplate readers were used: TECAN GENIos Pro, TECAN M200 Infinite Pro, and BioTek Synergy™ 2. The MTPs were incubated with orbital intermediate shaking at suitable temperature (37 °C ± 1 °C for *E. coli* and *B. subtilis*; 30 °C ± 1 °C for *S. cerevisiae*). The mixing performance and OTR were evaluated using the correlations suggested by Hermann et al. (2003), and an OTR of 7 mmol O_2_ L^−1^ h^−1^ was estimated for these operational conditions. The growth curves were determined by measuring the OD_600_ of each well every 15 min, during 24 h. All the measurements were performed within the linear OD range of the instrument. The data were exported from the microplate reader in ASCII format and further processed in Excel (Microsoft Office 2010).

### Modeling the microbial growth rates and lag-times

The maximum growth rate *μ*_max_ (h^−1^) and lag-time *λ* (h) are parameters typically used to characterize the kinetics of microbial growth. To assess these parameters, the lag-time model proposed by Baranyi and Roberts (1994) has been used:

1 $$ \frac{dx}{xdt}={\mu}_{\mathit{\max}}\alpha (t)f(x), with\;x\left(t=0\right)={x}_0 $$

In this model, *x*_0_ (g L^−1^) and *x* (g L^−1^) are the initial and actual cell densities, respectively, *t* (h) is the time, *α*(*t*) is the adjustment function delaying the transition from the lag-time to the exponential phase, and *f*(*x*) is the inhibition function defining the transition of the curve to the stationary phase. As only data from the lag-phase and exponential growth phase have been considered in the present work, the inhibition function, where oxygen limitation plays a fundamental role, can be omitted. The adjustment function has been defined according to the literature (Baranyi and Roberts 1994; Baty and Delignette-Muller 2004):

2 $$ \alpha (t)=\frac{q_0}{q_0+\mathit{\exp}\left(-{\mu}_{\mathit{\max}}t\right)\ } $$

where *q*_0_ quantifies the physiological viability of the inoculum for each specific environment. Baranyi and Roberts (1994) linked this parameter to *μ*_max_ and *λ* according to

3 $$ {q}_0={\left[ \exp \left({\mu}_{\mathit{\max}}\lambda \right)-1\right]}^{-1} $$

As a result, the solution for Eq. 1 is

4 $$ x(t)={x}_0\ \left[1+\mathit{\exp}\left({\mu}_{\mathit{\max}}\left(t-\lambda \right)\right)-\mathit{\exp}\left(-{\mu}_{\mathit{\max}}\lambda \right)\right] $$

Parameter estimation in Eq. 4 was performed by iterative nonlinear regression using Matlab 2013b (MathWorks). The parameter dependency and sensitivity of Matlab’s *lsqnonlin* function were inspected and minimized. The initial values for the parameters were chosen based on the experimental observations, and the measurement error in the initial cell density was tackled by estimating *x*_0_ along with *μ*_max_ and *λ*. The upper bound for the regression, i.e., the transition of the growth curves to the stationary phase, was chosen based on visual inspection accounting for all the curves belonging to each strain–*C*_*i*_ dataset. As a result, data points beyond the linear part of the logarithmic growth curves were excluded from the fit. Simultaneous optimization was performed for each curve by minimizing the sum of squared residuals. The parameter *q*_0_ was ultimately determined from *μ*_max_ and *λ* using Eq. 3. The average values of *μ*_max_, *λ*, and *q*_0_were determined for each dataset and finally compared using Welch’s unequal variance *t* test, with a significance level of 5 % (Welch 1938).

### Modeling the microbial tolerance to product inhibition

Several mathematical models have been proposed to quantify product inhibition kinetics, focusing mainly on the inhibiting effect of alcohols (Aiba et al. 1968; Dagley and Hinshelwood 1938), weak acids (Quintas et al. 2005), and ethyl esters (Urit et al. 2013) on different microorganisms. These models have been extensively reviewed elsewhere (Han and Levenspiel 1988; Mulchandani and Luong 1989; Urit et al. 2013). In the present work, three product-inhibition models (Table 1) were inspected, mainly for their simplicity and applicability regarding similar strains and inhibitors to those used herein.

**Table 1:** Product-inhibition models used to fit the experimental data

Type	Authors	Equation	Eq. no.
Linear	Dagley and Hinshelwood (1938)	${\mu}_{\mathit{\max}}={\mu}_{\mathit{\max},0}\left(1-\frac{C_i}{C_{\mathit{\max},i}}\right)$	(5)
Exponential	Aiba et al. (1968)	${\mu}_{\mathit{\max}}={\mu}_{\mathit{\max},0}\left[ \exp \left(-\frac{C_i}{K_{\mathit{\exp},i}}\right)\right]$	(6)
Hyperbolic	Quintas et al. (2005)	${\mu}_{\mathit{\max}}={\mu}_{\mathit{\max},0}{\left(1+\frac{C_i}{K_{hyp,i}}\right)}^{-1}$	(7)

*C*_*i*_ (g L^−1^) is the concentration of inhibiting agent in the fermentation medium; *μ*_max_ (h^−1^) is the maximum growth rate observed in the presence of each *C*_*i*_; *μ*_max , 0_ (h^−1^) is the maximum growth rate in the absence of inhibitor; and *C*_max , *i*_, *K*_exp , *i*_, and *K*_*hyp* , *i*_ are indicators of microbial tolerance, for which higher values denote a higher tolerance to the inhibitors. In the linear approach, *C*_max , *i*_ (g L^−1^) stands for the inhibitory threshold at which the microbial growth is completely inhibited, considering that 0 ≤ *C*_*i*_≤*C*_max , *i*_ (Dagley and Hinshelwood 1938). On the other hand, *K*_exp , *i*_ (g L^−1^) represents the inhibitory threshold in the exponential relation between the growth rate and the product concentration (Aiba et al. 1968) and *K*_*hyp* , *i*_ (g L^−1^) represents the inhibitor concentration at which half of the rate of substrate consumption is used for cell maintenance rather than growth, as described by Quintas et al. (2005) on the basis of cell energy requirements. Parameter estimation in Eqs. 5, 6, and 7 was performed by iterative nonlinear regression using the generalized reduced gradient (GRG) algorithm in Excel add-in Solver (Microsoft Office 2010). The initial values for the parameters were chosen based on the experimental observations, and the error in *μ*_max , 0_ was tackled by estimating this parameter along with *C*_max , *i*_, *K*_exp , *i*_, or *K*_*hyp* , *i*_. Simultaneous optimization was performed by minimizing the sum of weighted squared residuals (relative weighting), imposing the same *μ*_max , 0_ for the whole set of inhibitors regarding each strain. The goodness of the fit was assessed based on the standard error of the estimate for each case, *σ*_*i*_ (%), and the microbial tolerance to the inhibitors was ultimately compared using the indicators provided by the model with the lowest overall weighted standard error of the estimate, *σ*_*est*_ (%).

## Results

### Inhibition assays in shake flasks

**Fig. 1:**
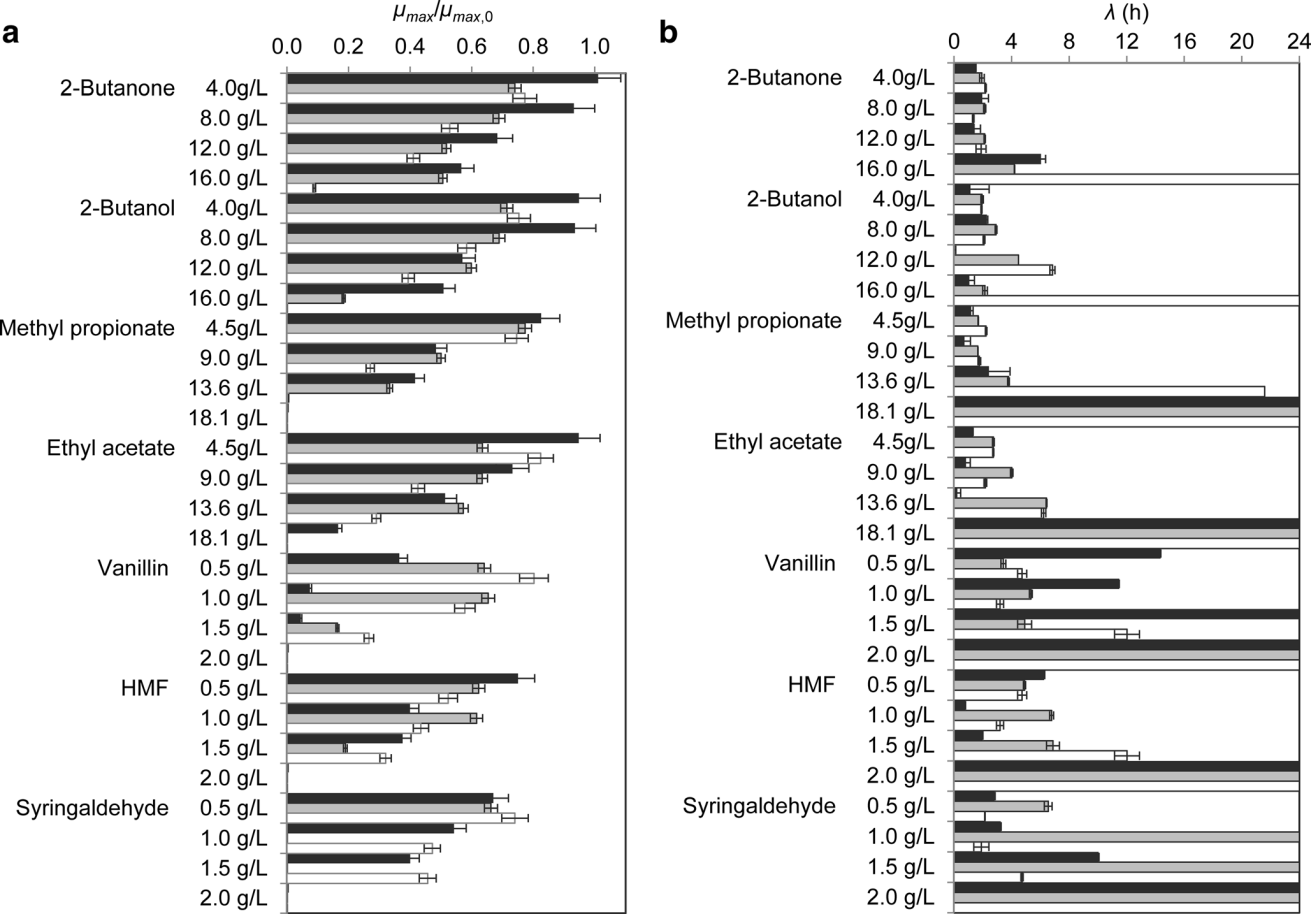
Kinetic parameters of microbial growth determined from inhibition assays in shake flasks with *S. cerevisiae* (*black boxes*), *B. subtilis* (*gray boxes*), and *E. coli* (*white boxes*) growing in defined mineral media containing different concentrations of inhibitors: **a** ratios of the maximum growth rates observed in the presence of inhibitors (*μ*_max_) to those observed in the absence of inhibitor (*μ*_max , 0_) and **b** lag-times (*λ*); *error bars* represent standard errors

Although shake flasks are widely used as less expensive bioreactors for multiple tasks, manual flask sampling has been proved to disturb cell growth (Büchs 2001). The sampling procedure was therefore limited to a sample every 2 h, allowing to gather sufficient data points to characterize the microbial growth. The concentration of volatile compounds in solution was consistent throughout the experiments, showing that no evaporation or microbial consumption occurred. Extreme cases were observed where none of the replicates grew at higher inhibitor concentrations, exhibiting extended lag-times (*λ*>24 h) and unquantifiable growth rates. This precluded parameter regression using Eq. 4 and further calculation of *q*_0_ in these cases. Apart from these occurrences, good fits were observed for the growth curves using the lag-time model. To facilitate the comprehension of the results, the maximum growth rates obtained for each strain–*C*_*i*_ dataset are presented as the ratio of *μ*_max_ to *μ*_max , 0_. This is shown in Fig. 1a. The regressed lag-times *λ* for each case are shown in Fig. 1b. The standard errors determined from two independent experiments are comparatively low, suggesting a good reproducibility (Fig. 1).

For all the cases investigated, the microbial growth displayed a slowing trend with increasing inhibitor concentrations. The majority of the cases displayed a virtually linear relation between *μ*_max_ and the inhibitor concentration, with the striking exception of *B. subtilis*, for which this is only observed when growing in medium containing methyl propionate. The statistical analysis showed that all the strains were significantly affected by the inhibitors at their lowest concentrations, with the exception of *S. cerevisiae*, which was not significantly affected by ethyl acetate at 4.5 g L^−1^ nor by 2-butanone or 2-butanol up to 8 g L^−1^. In these cases, a progressive inhibition of growth is suggested to occur with higher inhibitor concentrations. Strikingly, *S. cerevisiae* tolerated up to 18.1 g L^−1^ ethyl acetate and grew in the presence of 16 g L^−1^ 2-butanol with a relative growth rate of 50 %. In fact, this strain proved to have a higher tolerance for 2-butanol, methyl propionate, and ethyl acetate when compared to the other strains, as its growth rates were affected to a lesser extent by higher inhibitor concentrations. Although *B. subtilis* could also tolerate up to 16 g L^−1^ 2-butanol, the growth rate was only about 20 % of that without any inhibitor. Both *S. cerevisiae* and *B. subtilis* exhibited similar tolerance to 2-butanone up to 16 g L^−1^. Among the fermentation products, methyl propionate had the most severe impact, resulting in complete inhibition of all the strains when exposed to 18.1 g L^−1^. Regarding the lignocellulose-derived products, these revealed a high inhibitory activity, as none of the strains grew in product concentrations of 2.0 g L^−1^. *S. cerevisiae* and *E. coli* showed comparable tolerance regarding HMF and syringaldehyde, growing in concentrations up to 1.5 g L^−1^. On the other hand, *B. subtilis* could not grow in syringaldehyde concentrations higher than 0.5 g L^−1^. Vanillin was the most inhibiting for the yeast, reducing its growth rate by 95 % at 1.5 g L^−1^. Regarding the lag-times (Fig. 1b), although these were expected to increase with inhibiting concentrations, we failed to find a clear trend in the behavior of the strains. Longer *λ* (h) was indeed observed for all the strains when growing in the presence of increased concentrations of 2-butanone, methyl propionate, vanillin, and syringaldehyde. However, *B. subtilis* and *E. coli* were clearly more affected than yeast by 2-butanol, ethyl acetate, and HMF at high concentrations. Strikingly, *E. coli* presented *λ*>24 h for the highest concentrations of all the inhibitors tested, suggesting its higher sensitivity when compared to the other microbial hosts. Recalling Eq. 3, the parameters *μ*_max_, *λ*, and *q*_0_ are intertwined, and thus the physiological viability of a culture growing in a specific test condition depends on the growth rate and lag-time observed in that condition only. As a result, no direct relation was found between the inhibitor concentration and the values of *q*_0_ (data not shown).

### Inhibition assays in microtiter plates

**Fig. 2:**
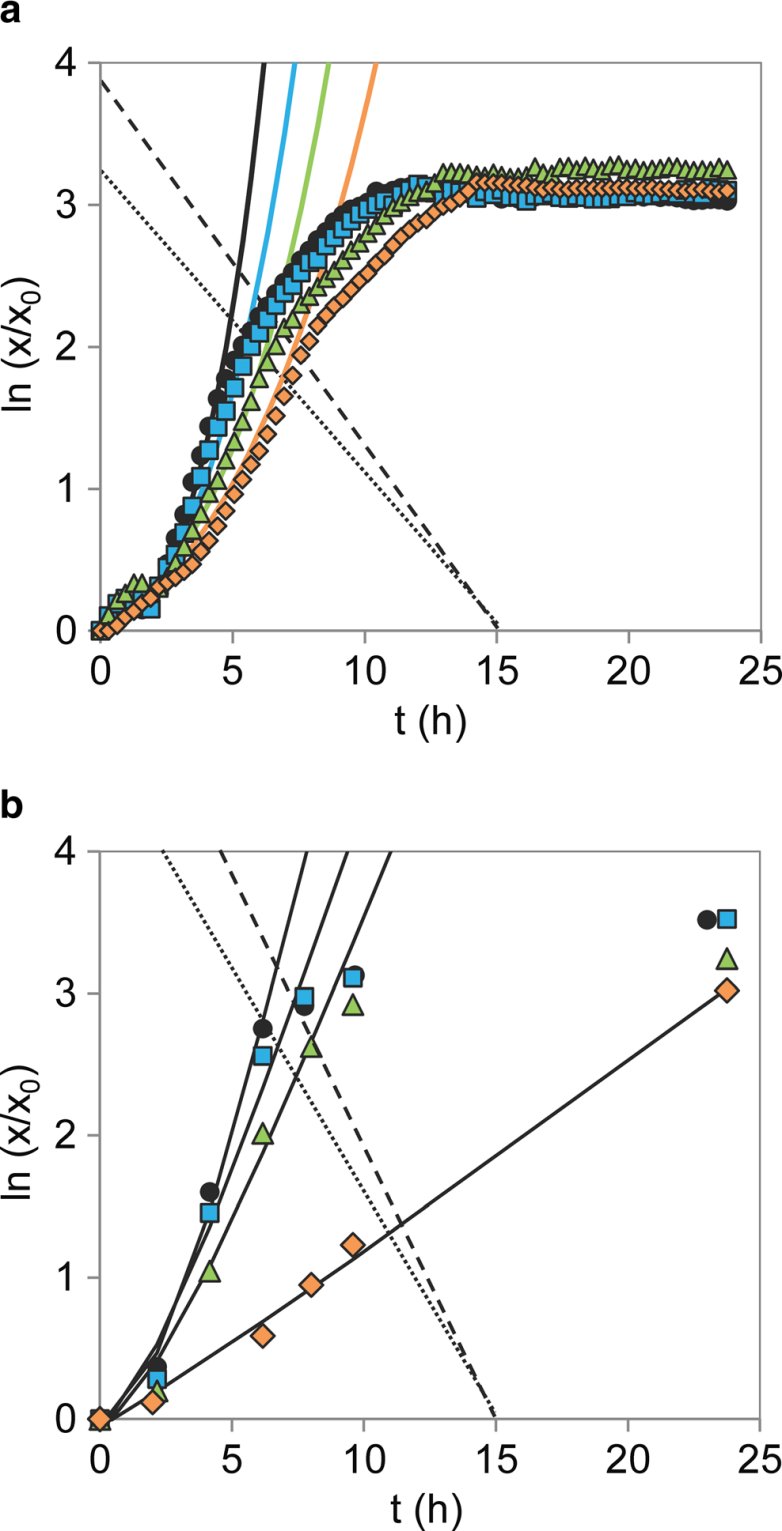
Comparative example of upper bounds in model fitting: logarithmic growth curves of *B. subtilis* measured in **a** MTPs and **b** shake flasks, in the absence (*circles*) and in the presence of 4 g L^−1^ (*squares*), 8 g L^−1^ (*triangles*), and 16 g L^−1^ (*diamonds*) 2-butanol; *markers* represent experimental data, *full lines* represent model predictions, *dotted line* represents upper bound chosen in the absence of 2-butanol, and *dashed line* represents upper bound chosen for the remaining conditions

Figure 2 shows some examples of worst-case model fittings for the growth of *B. subtilis* in different concentrations of 2-butanol, both in MTPs and shake flasks. It is clear from Fig. 2a that the cells experienced oxygen limitation in the MTPs, as the slopes of the curves declined slightly before the stationary phase was reached. This was not observed in the growth curves in shake flasks (Fig. 2b). The upper bounds chosen for the regression analysis of MTP data accounted for this observation, as shown by the dotted line representing the upper bound for the reference fermentation and the dashed line for the other conditions, in Fig. 2a. The same upper bound was used for different conditions in this particular case.

**Fig. 3:**
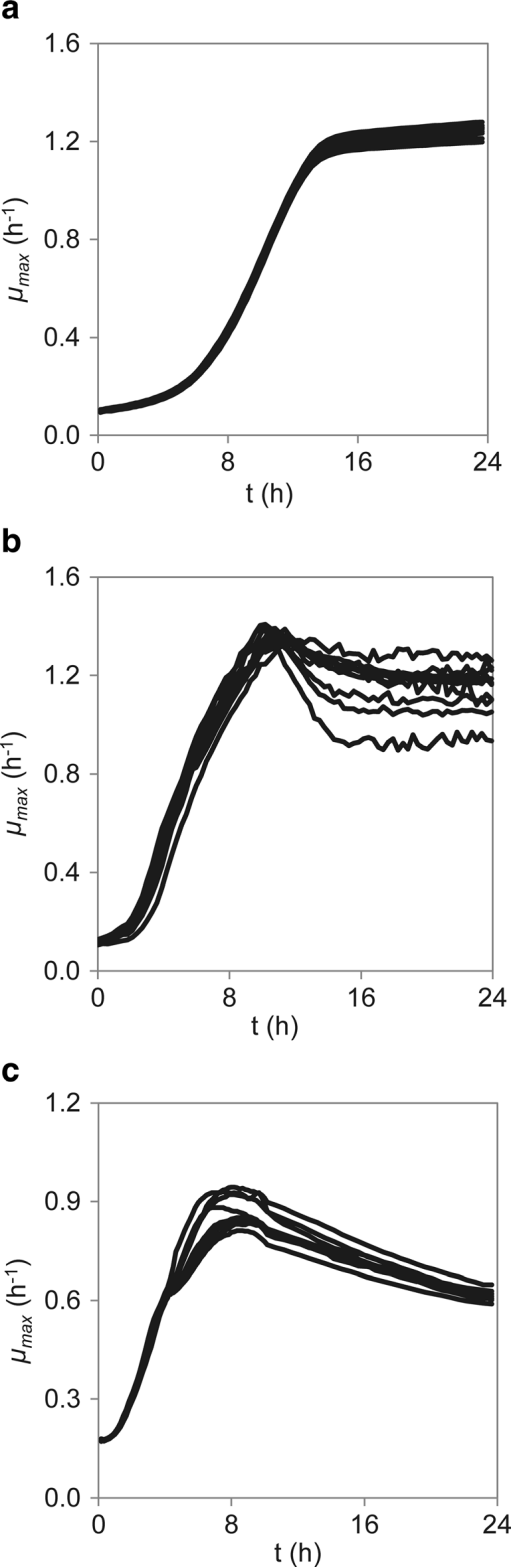
Experimental growth curves of **a** *S. cerevisiae*, **b** *B. subtilis*, and **c** *E. coli* in defined mineral media without inhibitor, measured in MTP with orbital intermediate shaking at suitable temperature (37 °C ± 1 °C for *E. coli* and *B. subtilis*; 30 °C ± 1 °C for *S. cerevisiae*)

In general, the experimental growth curves were well fitted by the simplified lag-time model, based on the average fitting deviation of 7.4 %. Nevertheless, great well-to-well disparity was observed for replicates within the same datasets, not only in the presence but also in the absence of inhibitors. The average variation found for *μ*_max_ was 13.7 % with a general increasing trend for increasing *C*_*i*_, much higher than that observed amid the replicates of shake flasks (5.4 %). As an attempt to verify the similarity between the assays, the average *μ*_max , 0_ values estimated for each strain were compared using Welch’s *t* test, and the outcome is presented in Table 2. The *μ*_max , 0_ values observed for *S. cerevisiae* and *B. subtilis* were identical in both assays at a significance level of 5 %, while the *μ*_max , 0_ values of *E. coli* were identical at a significance level of 10 %. This suggests that the cultivation conditions were fairly identical in both assays. However, when comparing the estimated *μ*_max_, considerable discrepancies were found, and the validation of the automated growth assay failed for the present case, as no correlation was found for any of the strains investigated. To address this matter, we investigated potential causes for well-to-well variability in the MTPs. In this work, the shaking mode was normalized for all the strains based on the calculated OTR, and identical growth rates were obtained for a minimum of 16 replicates growing in reference medium (Fig. 3), indicating the low impact of the shaking mode on the growth reproducibility. Noticeably, a significant variability was observed for *B. subtilis* and *E. coli* at higher cell densities (Fig. 3b, c, respectively), which was not observed in *S. cerevisiae*. Overall, *S. cerevisiae* had the lowest well-to-well variation, 9.5 %, while *B. subtilis* and *E. coli* had average variations of 15.3 and 15.9 %, respectively. This suggests that the operational temperature also plays a role in the reproducibility of the results in MTPs. In fact, the microtiter wells were clearly affected by evaporation and cell sedimentation, especially at 37 °C. As an attempt to determine the influence of evaporation on the optical density measurements, the optical density of water at 999 nm (OD_999_) was monitored at 37 °C during 24 h. The rates of evaporation were observed to vary strikingly depending on the well position, as the values of OD_999_ on the outer rows of the MTP decreased within a range of 15–100 %. Additionally, the evaporation of 2-butanol and methyl propionate, two volatile compounds with distinct boiling points of 100 and 80 °C, respectively, was examined at 30 and 37 °C. The evaporation rate of these compounds, *γ*_*i*_ (g L^−1^ h^−1^), was modeled according to *γ*_*i*_ = *k*_*s*_ *a* ∙ *C*_*i*_ (Truong and Blackburn 1984), where *k*_*s*_ *a* (h^−1^) is the evaporation rate constant. At 30 °C, the evaporation rate of 2-butanol was nearly unquantifiable after 24 h, while the *k*_*s*_ *a* value of methyl propionate was 0.0433 ± 0.0005 h^−1^. At 37 °C, the *k*_*s*_ *a* values increased significantly to 0.00638 ± 0.00002 h^−1^ for 2-butanol and 0.073 ± 0.003 h^−1^ for methyl propionate. It is clear that the evaporation rate not only depends on temperature but also on the volatile concentration and thus on the evaporation rate of water, varying with the well position in the MTP. Aware of these occurrences, attempts were made to prevent evaporation by filling the outer positions of the MTPs with distilled water and using special covers for 96 MTPs (Enzyscreen low-evaporation sandwich covers). Unfortunately, the results obtained at such conditions were analogous to the previous experiments. Another curious observation was the enhanced turbidity in wells containing higher concentrations of 2-butanone, methyl propionate, and ethyl acetate, which significantly increased the OD_600_ values in these cases. This was also observed in control wells without cells, indicating some sort of reactivity. Although these products have been reported as good solvents for polystyrene (Brown and Fundin 1991; Imre and Van Hook 1996), swelling and dissolution of the polymer is a slow process that has only been observed in the presence of pure compounds and thus it was not expected to happen in these dilute aqueous solutions.

**Table 2:** Maximum growth rates of *S. cerevisiae*, *B. subtilis*, and *E. coli* in defined mineral media without inhibitor (*μ*_max , 0_), determined from experimental shake flask and MTP data

	*μ*_max , 0_ (h^−1^)	
	SF	MTP
*S. cerevisiae*	0.34 ± 0.01	0.35 ± 0.06
*B. subtilis*	0.76 ± 0.02	0.8 ± 0.2
*E. coli*	0.7 ± 0.1	0.6 ± 0.1

### Microbial tolerance to product inhibition

**Fig. 4:**
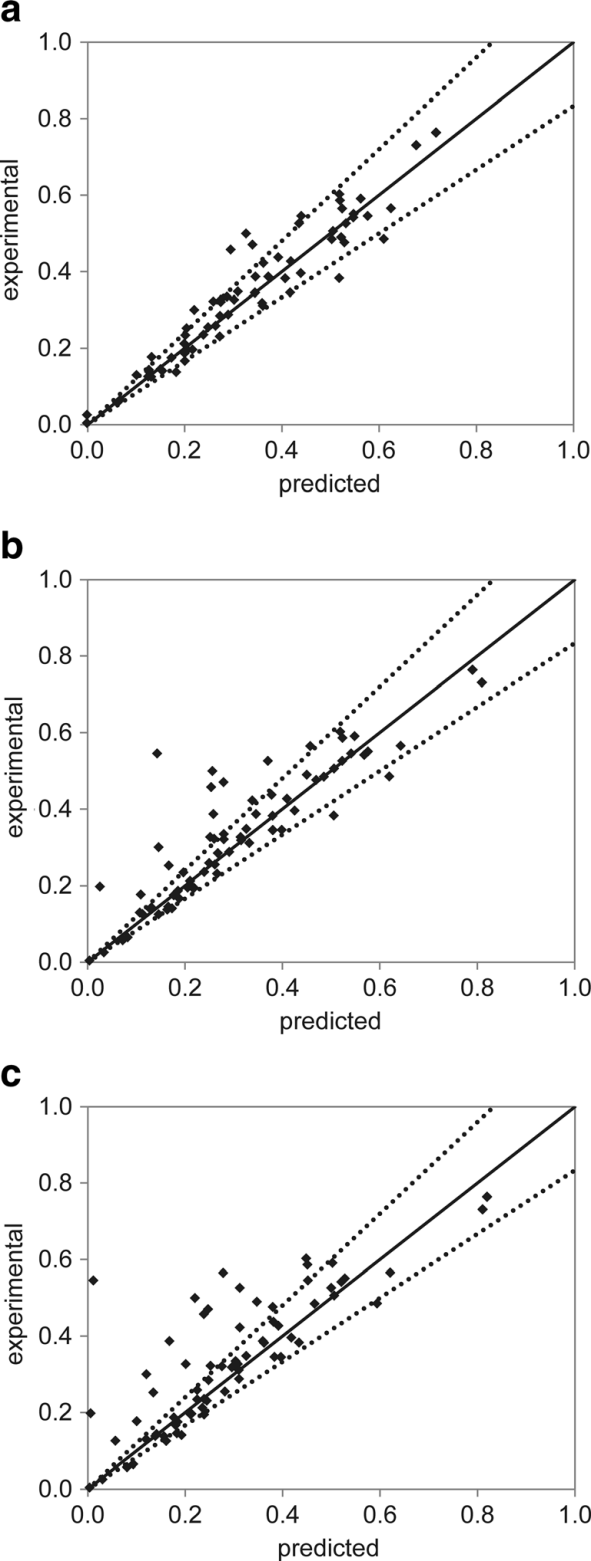
*Parity plot* showing the maximum growth rates (*μ*_max_) determined from experimental shake flask data, against those predicted by the **a** linear model, **b** exponential model, and **c** hyperbolic model; full line *x* = *y* added as reference; *dotted lines* represent a standard error of ±20 %

Due to the perceived uncertainty in the MTP assay, the tolerance of each strain to the inhibitors was assessed using the shake flask data exclusively. The correlation between the experimental data and the predicted values of *μ*_max_ using the product-inhibition models described in Eqs. 5, 6, and 7 is shown in Fig. 4, accounting for all the strains and inhibitors investigated. A good correlation was found between the observed microbial growth rates of the strains and those estimated using the linear model (Fig. 4a). Comparatively, and although the majority of the observations lies within a ±20 % deviation, the exponential and the hyperbolic models (Fig. 4b, c, respectively) under-predicted the microbial growth for a considerable number of cases. The predictability of the models for each inhibitor was inspected by looking at the overall standard errors of the estimates, *σ*_*est*_ (%), accounting for the individual fits obtained for each strain. The results are presented in Table 3 and also depict what is shown in Fig. 4: the model proposed by Dagley and Hinshelwood (1938) exhibited the lowest standard errors and allowed better predictions for the effect of all the inhibitors on the strains. Two-parameter models have been reported to outperform the simple one-parameter models tested herein, when describing the growth inhibition of *K. marxianus* by ethyl acetate (Urit et al. 2013). These models account for an additional regression parameter *n*, whose magnitude determines whether the inhibition trend is linear (*n* = 1), progressive (*n* >1), or declining (*n* <1). For the sake of comparison, the experimental shake flask data were fitted using the progressive model proposed by Luong (1985). The parameter estimation and goodness of fit were assessed using the same approach as previously described. The standard errors of the estimates found when fitting the data with the progressive model were slightly lower, but analogous to those determined for the simpler linear model. The fit of both models was thus compared by means of a model reduction test (*F* test), where the improvement of adding the extra parameter in the progressive model was quantified as the difference in the resulting sum of squares. The *p* values calculated using this approach were much higher than the traditional value of 0.05 (0.8, 0.6, and 0.5 for the fittings of *B. subtilis*, *E. coli*, and *S. cerevisiae*, respectively), showing that it is not statistically significant to add a parameter and thus complexity to the product-inhibition model used. Based on these observations, the indicators of microbial tolerance *C*_max , *i*_ estimated using the linear model were chosen for further comparison among the strains. The results are depicted in Table 4, where the individual standard errors of the estimates *σ*_*i*_ (%) are also presented. All the estimates show standard errors lower than 20 %, with rare exceptions: the effect of vanillin on the growth of *S. cerevisiae* and the effect of HMF on the growth of *E. coli*, which were better described by the exponential model (*σ*_*i*_= 15.4 %) and hyperbolic model (*σ*_*i*_= 10.9 %), respectively.

**Table 3:** Overall standard error of the estimates *σ*_*est*_ (%) for the predictions by each product-inhibition model; the lowest σ_est_ (%) indicate the best fits to shake flask experimental data

	*σ*_*est*_ (%)		
Model Eq. no.	Linear (5)	Exponential (6)	Hyperbolic (7)
2-Butanone	11.8	20.0	29.0
2-Butanol	13.9	17.9	22.7
Methyl propionate	10.8	34.4	41.2
Ethyl acetate	16.2	20.5	27.0
Vanillin	17.8	19.9	31.2
HMF	18.0	19.2	20.0
Syringaldehyde	10.4	12.1	10.5

**Table 4:** Inhibitor concentrations at which the microbial growth is completely inhibited (*C*_max , *i*_), estimated from experimental data (shake flasks) using the linear product-inhibition model; σ_i_ (%) are the standard errors of the estimates

	*S. cerevisiae*	*σ*_*i*_ (%)	*B. subtilis*	*σ*_*i*_ (%)	*E. coli*	*σ*_*i*_ (%)
2-Butanone	45 ± 17	11.4	31 ± 6	9.1	17.8 ± 0.4	14.4
2-Butanol	36 ± 9	12.6	20 ± 1	18.7	21 ± 3	6.5
Methyl propionate	23 ± 5	11.6	21 ± 2	6.0	13.68 ± 0.02	13.4
Ethyl acetate	22 ± 1	19.6	30 ± 8	14.6	19 ± 2	12.6
Vanillin	1.08 ± 0.02	22.9	1.84 ± 0.08	18.3	2.2 ± 0.2	12.0
HMF	2.2 ± 0.3	18.0	1.9 ± 0.1	15.7	2.2 ± 0.2	20.1
Syringaldehyde	2.5 ± 0.5	8.2	2 ± 1	6.0	2.7 ± 0.4	13.7

Based on the Welch’s test results, *S. cerevisiae* and *B. subtilis* are significantly more tolerant than *E. coli* to 2-butanone, methyl propionate, and ethyl acetate. In fact, the thresholds predicted for *S. cerevisiae* are higher than those of *B. subtilis* for all the fermentation products, with the exception of ethyl acetate. On the other hand, the threshold concentrations found for the lignocellulose-derived products are comparable regarding all the strains, although *E. coli* is slightly more tolerant to vanillin. Interestingly, when comparing the data in Table 4 with the observations presented in Fig. 1, the thresholds predicted for *B. subtilis* regarding ethyl acetate appear to be overestimated, as this strain was unable to grow in 18 g L^−1^ ethyl acetate. This might be justified by an apparent lag of inhibition at low ester concentrations, which has also been observed for *K. marxianus* (Urit et al. 2013). Overall, the results in Table 4 are in agreement with previous studies reporting that inhibition thresholds strongly depend on the microbial strain and inhibitor tested (van der Pol et al. 2014).

## Discussion

### The limited applicability of microtiter plates in the present study

In a growth tolerance assay, the inhibitors in the fermentation medium impose a continuous stress on the cells. Under these circumstances, the cells are expected to adapt while growing, which leads to a differential expression of the genes required for growth, and thus great variation is expected in latency times and maximum growth rates (Gonzalez-Ramos et al. 2013; Swinnen et al. 2014). However, in the present work we noticed that the variance found in the MTP assay, not observed in the shake flasks, was mostly related to technical issues instead of intraspecies variability. Many advances have been reported concerning MTP bioreactors for rapid and reliable bioprocess development (Büchs 2001; Funke et al. 2010; Jung et al. 2015; Klockner and Buchs 2012). However, as cells grow under suboptimal conditions, such as inhibiting environments, they might experience apoptosis resulting in cell adhesion and aggregates, ultimately disturbing the cell density measurements (Reinhart et al. 2015). To reduce cell sedimentation and enhance growth reproducibility, the shaking mode of MTP readers has been optimized by some researchers (Jung et al. 2015; Warringer and Blomberg 2003). The optimal shaking mode varied depending on the strain (Warringer and Blomberg 2003), and good reproducibility has only been achieved when intermittent shaking modes were used (Jung et al. 2015; Warringer and Blomberg 2003). This leads to different operational conditions for each strain, which becomes unfeasible when several strains are to be compared under the same circumstances. The evaporation observed in this work also promoted a trendless well-to-well variability. Gonzalez-Ramos et al. (2013) reported 50 % 1-butanol evaporation in unsealed MTPs and reduced this to 10 % by sealing the plates with a gas-impermeable film that also prevented aeration. In this case, a microaerobic instead of a fully anaerobic environment was desired, which led us to avoid this approach. Despite the efforts to correct the optical density based on water evaporation, great discrepancy was still observed. The vapor pressures of aqueous mixtures of 2-butanone, 2-butanol, methyl propionate, and ethyl acetate are higher than those of the pure compounds, depending on the temperature and mixture concentration at ambient pressure. Thus, the evaporation rates of water and inhibitors varied with the concentrations investigated and working temperatures in the MTPs. While the volatile products would easily evaporate along with water resulting in unknown medium concentrations, the lignocellulose-derived inhibitors would become more concentrated as water evaporated, leading to their sedimentation along with the dead cells. None of these issues was observed in the shake flasks, since these could be properly sealed without compromising microaeration. Given all these reasons, and opposed to what has been achieved by other researchers (Chaturvedi et al. 2014; Huber et al. 2009; Quintas et al. 2005), the observations made in shake flask tests could not be fairly validated in MTPs. The datasets provided by the MTPs facilitated the model fitting and parameter estimation with small residuals and errors; however, the technical issues encountered could have led to severe misinterpretation of the collected data.

### Quantification of inhibition on microbial growth rates

The simplified lag-time model proved to be a useful and reliable tool to describe the experimental growth curves of the strains in the presence of all the inhibitors investigated. Similarly to what has been previously reported in microbial growth studies (Swinnen et al. 2014), no linear correlation was found between *μ*_max_ and *λ* for any of the strains (*R* ^2^ = 0.66 for *S. cerevisiae*, *R* ^2^ = 0.76 for *E. coli*, and *R* ^2^ = 0.72 for *B. subtilis*). As a result, no correlation was found between the parameters and the physiological viability of the inoculum. It is known that the lag-phase estimation is greatly influenced by the technique used to monitor bacterial growth (Baty and Delignette-Muller 2004). In the present case, the observed lag-phase duration results not only from the adaptation of the microbial hosts to the adverse environment but also from the death of a fraction of cells that could not survive the inhibition, followed by growth of the enduring cells. The OD growth measurements accounted for both living and dead cells, which can mask the lag-phase duration. This fact is supported by Swinnen et al. (2014), who reported that the actual lag of enduring cells is significantly shorter than that detected by OD measurements.

The linear product-inhibition model was an important tool to predict critical concentrations of inhibitors, allowing a fair comparison of inhibitory thresholds amid the strains investigated. The exponential and hyperbolic models, on the other hand, tended to predict a declining inhibition with increasing inhibitor concentrations. In the present work, this trend has been mostly observed for *S. cerevisiae* and *E. coli* growing in medium containing vanillin and HMF, respectively. A progressive model could have also been used to predict the effect of the inhibitors on the strains, but we proved that the results were not significantly improved by this approach. Overall, the results showed that *S. cerevisiae* and *B. subtilis* are comparatively more tolerant than *E. coli* to the fermentation products tested, namely, 2-butanone, methyl propionate, and ethyl acetate. *S. cerevisiae* revealed the highest critical concentration for 2-butanol, which might be explained by the fact that this strain is a spontaneous mutant resulting from an evolved population growing under increased 1-butanol concentrations, which could also grow in approximately 3 % (*v*/*v*) 2-butanol (Gonzalez-Ramos et al. 2013). The threshold estimated for 2-butanol in the present study, 36 ± 9 g L^−1^, is in agreement with this observation. Methyl propionate had a severe impact on the strains, resulting in complete inhibition of all the strains when exposed to the highest concentration tested. This is clear from Table 4, where the critical concentration values have been estimated as 23 ± 5, 21 ± 2, and 13.68 ± 0.02 g L^−1^ for *S. cerevisiae*, *B. subtilis*, and *E. coli* respectively. Strikingly, the thresholds determined for ethyl acetate were higher than that previously reported for *K. marxianus* (17 g L^−1^) (Urit et al. 2013). Despite the fact that ethyl acetate is an isomer of methyl propionate, the strains were slightly more tolerant to this ester, which is also demonstrated by the values in Table 4. This might be related to the different hydrophobicity of the isomers, since it has been suggested that more hydrophobic compounds would be expected to easily permeate microbial membranes, exhibiting an increased toxicity (Zaldivar and Ingram 1999). In fact, methyl propionate exhibits a more hydrophobic behavior, since its log*P*_*oct*/*water*_ is higher than that of ethyl acetate (0.82 versus 0.73, respectively) (Lide 2004; Smallwood 1996). The fact that certain organisms are able to metabolize ethyl acetate, namely, *H. anomala* (Tabachnick and Joslyn 1953), might also enhance the microbial tolerance to this inhibitor. Nevertheless, our observations are insufficient to explain this behavior herein.

Among the lignocellulosic degradation products tested, vanillin had the most severe effect on yeast, which could barely tolerate 1.5 g L^−1^ of inhibitor. Although this observation is in agreement with what has been previously reported by Delgenes et al. (1996), the yeast tolerance observed for syringaldehyde in the present study is remarkably higher than that reported by the same author: 40 % of the reference growth rate against 19 % for a concentration of 1.5 g L^−1^. All the strains depicted analogous values for the threshold concentrations regarding HMF and syringaldehyde. However, and opposed to what has been reported (van der Pol et al. 2014), none of the strains investigated in this work showed significant growth in medium containing lignocellulosic product concentrations of 2.0 g L^−1^.

Overall, based on the observed growth rates and lag-times, *S. cerevisiae* was slightly more tolerant than the other strains to the majority of the inhibitors, having great potential to be engineered and further established as host for the bio-based production of methyl esters. Even though *B. subtilis* showed similar tolerance to some of the inhibitors investigated, namely, 2-butanone, methyl propionate, ethyl acetate, HMF, and syringaldehyde, the lag-times observed were recurrently higher than those of *S. cerevisiae*. Additionally, it must be recalled that this strain is not as robust in microaerobic environments (Cruz Ramos et al. 2000). The *S. cerevisiae* IMS0351 used in the present study proves that evolutionary methods, such as natural selection and evolutionary dynamics, are highly valuable to improve inhibitor tolerance (Gonzalez-Ramos et al. 2013). Repetitive growth under increasing inhibitor concentrations might be a promising and effective technique to further enhance inhibitor tolerance in *S. cerevisiae* IMS0351 and accelerate the methyl propionate bio-based production.
